# Creating a population-based cohort of children born with and without congenital anomalies using birth data matched to hospital discharge databases in 11 European regions: Assessment of linkage success and data quality

**DOI:** 10.1371/journal.pone.0290711

**Published:** 2023-08-30

**Authors:** Maria Loane, Joanne E. Given, Joachim Tan, Ingeborg Barišić, Laia Barrachina-Bonet, Clara Cavero-Carbonell, Alessio Coi, James Densem, Ester Garne, Mika Gissler, Anna Heino, Sue Jordan, Renee Lutke, Amanda J. Neville, Ljubica Odak, Aurora Puccini, Michele Santoro, Ieuan Scanlon, Stine K. Urhoj, Hermien E. K. de Walle, Diana Wellesley, Joan K. Morris

**Affiliations:** 1 Faculty of Life and Health Sciences, Ulster University, Belfast, Northern Ireland, United Kingdom; 2 Population Health Research Institute, St George’s University of London, London, United Kingdom; 3 Children’s Hospital Zagreb, Centre of Excellence for Reproductive and Regenerative Medicine, Medical School University of Zagreb, Zagreb, Croatia; 4 Rare Diseases Research Unit, Foundation for the Promotion of Health and Biomedical Research in the Valencian Region, Valencia, Spain; 5 Unit of Epidemiology of Rare Diseases and Congenital Anomalies, Institute of Clinical Physiology, National Research Council, Pisa, Italy; 6 Biomedical Computing Limited, Battle, United Kingdom; 7 Department of Paediatrics and Adolescent Medicine, Lillebaelt Hospital, University Hospital of Southern Denmark, Kolding, Denmark; 8 Department of Knowledge Brokers, THL Finnish Institute for Health and Welfare, Helsinki, Finland; 9 Faculty of Medicine, Health and Life Sciences, Swansea University, Swansea, Wales, United Kingdom; 10 Department of Genetics, University Medical Center Groningen, University of Groningen, Groningen, The Netherlands; 11 Emilia Romagna Registry of Birth Defects, Center for Clinical and Epidemiological Research, University of Ferrara, Azienda Ospedaliero- Universitaria di Ferrara, Ferrara, Italy; 12 Territorial Care Service, Emilia Romagna Health Authority Bologna, Bologna, Italy; 13 Public Health Wales, Swansea, United Kingdom; 14 Section of Epidemiology, University of Copenhagen, Copenhagen, Denmark; 15 Wessex Clinical Genetics Service, Princess Anne Hospital, Southampton, United Kingdom; Haramaya University Faculty of Health Sciences: Haramaya University College of Health and Medical Sciences, ETHIOPIA

## Abstract

Linking routinely collected healthcare administrative data is a valuable method for conducting research on morbidity outcomes, but linkage quality and accuracy needs to be assessed for bias as the data were not collected for research. The aim of this study was to describe the rates of linking data on children with and without congenital anomalies to regional or national hospital discharge databases and to evaluate the quality of the matched data. Eleven population-based EUROCAT registries participated in a EUROlinkCAT study linking data on children with a congenital anomaly and children without congenital anomalies (reference children) born between 1995 and 2014 to administrative databases including hospital discharge records. Odds ratios (OR), adjusted by region, were estimated to assess the association of maternal and child characteristics on the likelihood of being matched. Data on 102,654 children with congenital anomalies were extracted from 11 EUROCAT registries and 2,199,379 reference children from birth registers in seven regions. Overall, 97% of children with congenital anomalies and 95% of reference children were successfully matched to administrative databases. Information on maternal age, multiple birth status, sex, gestational age and birthweight were >95% complete in the linked datasets for most regions. Compared with children born at term, those born at ≤27 weeks and 28–31 weeks were less likely to be matched (adjusted OR 0.23, 95% CI 0.21–0.25 and adjusted OR 0.75, 95% CI 0.70–0.81 respectively). For children born 32–36 weeks, those with congenital anomalies were less likely to be matched (adjusted OR 0.78, 95% CI 0.71–0.85) while reference children were more likely to be matched (adjusted OR 1.28, 95% CI 1.24–1.32). Children born to teenage mothers and mothers ≥35 years were less likely to be matched compared with mothers aged 20–34 years (adjusted ORs 0.92, 95% CI 0.88–0.96; and 0.87, 95% CI 0.86–0.89 respectively). The accuracy of linkage and the quality of the matched data suggest that these data are suitable for researching morbidity outcomes in most regions/countries. However, children born preterm and those born to mothers aged <20 and ≥35 years are less likely to be matched. While linkage to administrative databases enables identification of a reference group and long-term outcomes to be investigated, efforts are needed to improve linkages to population groups that are less likely to be linked.

## Introduction

Congenital anomalies are structural anomalies and genetic syndromes that occur during development of the embryo and affect 2–3% of all children born in Europe every year. They are a major cause of perinatal and infant morbidity and disability in children [1–3], yet little is known on the long-term outcomes of these children. The European surveillance of congenital anomalies (EUROCAT) network of population-based registries provides essential epidemiologic information and surveillance of congenital anomalies in Europe, but information is mainly collected up to a baby’s first year of life, and few registries record information on control children. The availability of regional or national administrative databases holding routinely collected healthcare information opens up the potential of using these data sources for research on long-term morbidity outcomes in children, or indeed other population groups, as well as the opportunity for identifying a control (reference) group. However, as administrative data were not primarily collected for research purposes, linkage success and the quality of the matched data must firstly be assessed to determine if they are fit for research purposes.

The EUROlinkCAT project (http://www.EUROlinkCAT.eu/home) aimed to investigate health and educational outcomes of European children with congenital anomalies in the first 10 years of life by linking to regional or national mortality, hospital discharge, prescription and educational databases [4]. All EUROCAT registries were invited to participate in the study, but some did not reply to the invite, or were unable to get study approval, or did not have the time or resources to commit to the study or did not have the necessary linkage infrastructure such as unique identifiers to link children across different databases. Standardised data (i.e. variables coded to a common specification) on congenital anomalies are already available from the EUROCAT registries [5]. The quality of linkage to national/vital statistics and mortality records to provide survival estimates for children with congenital anomalies in 18 EUROCAT registries in 13 countries has been described previously [6]. The aim of this study is to describe the rates of linking children with and without congenital anomalies to regional or national hospital databases for use in the EUROlinkCAT morbidity studies, and to evaluate the quality of the matched data.

## Materials and methods

### Population

This EUROlinkCAT morbidity cohort study includes children born with major congenital anomalies from 11 European population-based congenital anomaly registries in 7 countries (Table 1). Data on children with congenital anomalies were extracted from the EUROCAT central database for ten registries and linked to vital statistics, whereas a specific script was produced to create the EUROCAT anomaly subgroups for Finland, as Finland only sends aggregate data to EUROCAT. Data on reference children (i.e. all children without congenital anomalies born in the region covered by the registry) were obtained from national/regional vital statistics (birth records) which included information on maternal and baby characteristics. Tuscany included a random sample of 10% of their population as reference children (matched with EUROCAT children on sex and year of birth), while the Northern Netherlands included 20% of their population as reference children (matched with EUROCAT children on year of birth). No reference population was available for the three English registries (East Midlands & South Yorkshire, Thames Valley and Wessex) as separate ethics permissions could not be obtained within the project timescale to link data on reference children. Zagreb selected 2–3 reference children from hospital maternity records for each child with a congenital anomaly, but these were later dropped from analyses as they were hospital-based, rather than population-based.

**Table 1:** Linked hospital databases, birth years included in the study, reference population and coding classification systems, by registry.

	Linked hospital databases ^a^	Birth years included in the study	Reference population (%)	Age of children (years) ^b^	Coding classification in hospital database	Surgery coding classification
Croatia, Zagreb	Republic of Croatia Bureau of Statistics or Croatian Health Insurance System	2008–2014	-	0–7	ICD-10	-
Denmark, Funen	The Danish National Patient Registry (DNPR)	1995–2014	100	0–9	ICD-10	Danish version of NCSP
Finland	Finnish Hospital Discharge Register	1997–2014	100	0–9	ICD-10	Finnish version of NCSP
Italy, Emilia Romagna	Hospital Discharge Data (SDO), Certificate of assistance at birth (CedAP)	2008–2014	100	0–7	ICD-9-CM	ICD-9-CM
Italy, Tuscany	Hospital Discharge Data (SDO and CedaP)	2005–2014	10	0–9	ICD-9-CM	ICD-9-CM
The Netherlands, North (LMR)	Dutch Hospital Data (Landelijke Medische Registratie)	1995–2010	20	0–9	ICD-10	-
The Netherlands, North (LBZ)	Dutch Hospital Data (Landelijke Basisregistratie Ziekenhuiszorg)	2013–2014	20	0–4	ICD-10	-
Spain, Valencian Region	Hospital Discharge Records	2010–2014	100	0–5	ICD-9-CM	ICD-9-CM
UK, Wales	Patient Episode Database for Wales	1998–2014	100	0–9	ICD-10	OPCS-4
UK, England, East Midlands & South Yorkshire	Hospital Episode Statistics	2003–2012	-	0–9	ICD-10	OPCS-4
UK, England, Thames Valley	Hospital Episode Statistics	2005–2013	-	0–9	ICD-10	OPCS-4
UK, England, Wessex	Hospital Episode Statistics	2004–2014	-	0–9	ICD-10	OPCS-4

^a a^ Includes information on hospital admissions/ discharges, diagnosis, surgery, ICU and ventilation use

^b^ Includes at least one year of follow-up

- No data

ICD = International Classification of Diseases; ICD-CM = International Classification of Diseases–Clinical Modification

NCSP = NOMESCO (Nordic Medico-Statistical Committee) Classification of Surgical Procedures

OPCS = Office for population Censuses and Surveys

Children born alive at ≥23 weeks gestation between 1^st^ January 1995 and 31^st^ December 2014 in the areas surveyed by the EUROCAT registries were followed up to their 10^th^ birthday or to the study end date (31^st^ December 2015), whichever was earlier, so that each child in the cohort had at least one year of follow-up information. Eight registries had information up to nine years of age for some children, while the maximum age of children in the other registries/data sources ranged from 4–7 years depending on the first year of the birth cohort included in the study (Table 1).

The Northern Netherlands changed their hospital database system during the study period, hence there were two data sources. LMR (Landelijke Medische Registratie) was used for children born 1995–2010, with at least one year follow-up to 2011, and LBZ (Landelijke Basisregistratie Ziekenhuiszorg) was used for children born 2013–2014, with follow-up to the end of 2017.

### Standardisation

**Fig 1:**
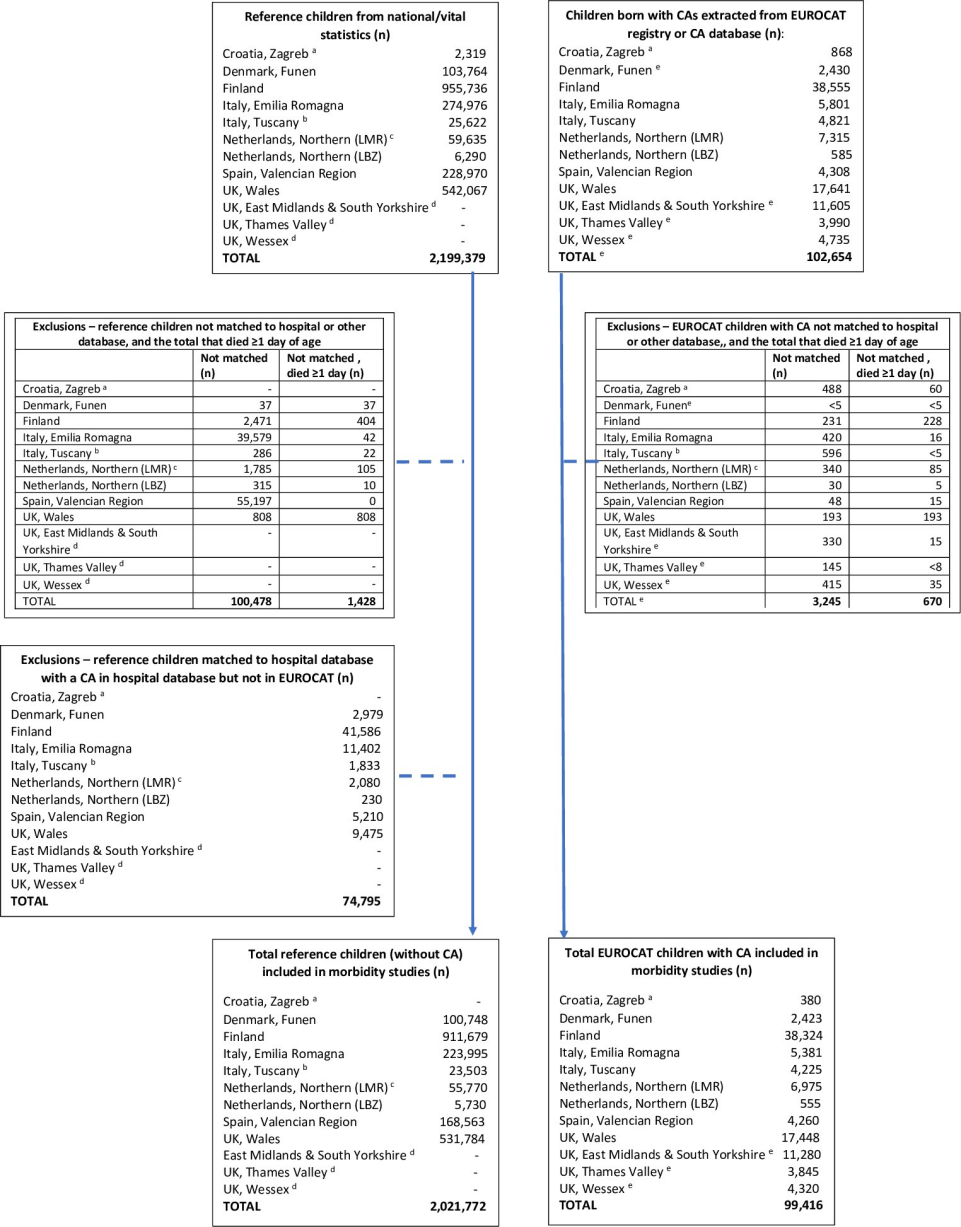
Flowchart showing exclusions and final study sample of children with congenital anomalies and reference children in each registry. ^a^ Zagreb reference children and children with congenital anomalies dropped from study. ^b^ Tuscany reference children = 10% of population. ^c^ NNL LMR and LBZ reference children = 20% of population; totals rounded to 0, 5 or 10. ^d^ English registries do not have reference children.^e^ Totals rounded. NB: Total children extracted from national/vital statistics and EUROCAT registries minus the exclusions do not add up to the total included in the morbidity study due to 1) rounding and 2) reference children in Zagreb were dropped from analysis (n = 2,319).

Throughout Europe, a variety of coding classification systems are used to code diagnoses and surgical procedures in hospital discharge databases (Table 1). This information is not standardised, hence a common data model (CDM) was developed to standardise data recorded in vital statistics and hospital discharge databases (S1 Table). When standardising the linked datasets in each region, reference children with a major or minor congenital anomaly (i.e. any ICD-10 code in the Q-chapter) [7] recorded in the hospital database were excluded from the reference population as these children may only have had an isolated minor anomaly which are not considered EUROCAT cases [4] or because of over-recording of congenital anomalies in hospital databases [8], or they may have congenital anomalies not recorded in the EUROCAT registry (Fig 1). These exclusions were necessary as an aim of the morbidity study was to assess the risk of hospitalisation and length of hospital stays in children with and without congenital anomalies, hence the importance of ensuring that reference children did not have any anomalies which may bias the results. In addition, as the majority of children in Europe are born in hospital, obstetric stays (defined using ICD-9 codes V30-V39 and ICD-10 codes Z37-Z39) were excluded. If the baby is transferred to an intensive care unit (ICU) or other hospital, these babies are included in the study.

### Methods of linkage

Electronic linkage was performed in 10 of the 11 regions, while one registry Zagreb, performed manual linkage using the child’s unique identification (ID) number. Five registries used “deterministic linkage” defined as a rule-based linkage typically requiring exact agreement on an ID number which uniquely identifies each individual in a country. One registry (Emilia Romagna) used “probabilistic linkage” defined as using match weights representing the likelihood of two records belonging to the same subject given agreement on a set of partial identifiers such as name, address, date of birth, gender, mother’s date of birth and residence to match cases [9]. The four UK registries used a combination of deterministic and probabilistic methods. Information on the type of identifiers used to link in each region have been published earlier [6]. Detailed information on the linkage methods and the identifiers used in deterministic and probabilistic linkage in each region/country are available in a EUROlinkCAT manual [10].

### Inclusion and exclusion criteria

To ensure that children with no hospital admissions during the study period were not “missed” linkages, registries were asked to link the children to hospital databases or other administrative databases such as vital statistics (e.g. death records), prescription databases or outpatient records inside and outside the study period. Children not matched to hospital discharge databases or other databases during the study period but matched outside the study period or after their 10^th^ birthday were included in the study population. In this study, children who were linked to any administrative database (hospital discharge databases or other databases) at any point within or outside the study period are referred to as matched children.

Children not matched to hospital discharge databases or other databases inside or outside the study period were excluded from the study population as they were assumed to be “missed” linkages (Fig 1). Children who died ≥1 day after birth not matched to any database inside or outside the study period were also excluded, as these may indicate that matches to hospital databases were missed as deaths in childhood are generally expected to be preceded by hospital admissions, with the exception of sudden infant death, murder and accidents (Fig 1).

### Data quality

For each registry, the Ulster University (UU) research team reported on the percentage of children with complete information on key variables relating to hospital stays (extracted using the standardised common data model) including dates of hospital admission/ discharge known, diagnosis for hospital stays, at least one admission to ICU, ventilator use, and dates and codes for surgical procedures. This information was confirmed by all registries. Two registries (Wales and the Northern Netherlands) had no data on ICU. The Northern Netherlands also had no data on ventilator use or surgery for this study due to sub-optimal recording of these data. The proportions of children with ICU admissions and ventilator use are calculated out of all children with a hospital admission.

### Statistical analysis

Researchers from St George’s, University of London (SGUL) developed a central linkage quality syntax script which was distributed to all registries to assess linkage success i.e. the likelihood of being matched. In addition, the central script compared characteristics of children included with those excluded from the study population to see if specific characteristics were associated with a greater likelihood of missed matches for children with congenital anomalies and reference children.

The odds of being matched to hospital discharge or other databases inside or outside the study period were examined by fitting univariate logistic regression models. Maternal and child characteristics (maternal age, baseline group was mothers aged 20–34 years; multiple births, baseline was singletons; infant sex, baseline was males; gestational age, baseline was ≥37 weeks; and birth weight, baseline was 2,500–3,999 grams) were included in the models as independent variables. Odds ratios (OR) and ORs adjusted by region were estimated for children with congenital anomalies only and reference children only.

### Accuracy of information recorded in linked datasets

The accuracy of recording the five above-mentioned maternal and child characteristics was assessed by comparing the EUROCAT variables with the equivalent variables in the linked datasets for children with congenital anomalies only. For example, the EUROCAT variable “SEX” of child was compared to the variable for sex in the linked file “L_CH_SEX”. All variables in the linked file were prefixed with L_ to differentiate these from the EUROCAT variables. The EUROCAT data were deemed to be the “gold standard” as these data are derived from multiple sources [5]. It was not possible to assess the recording accuracy of these variables for reference children, as there was no gold standard to compare with.

### Reporting restrictions

As previously described [4, 6], six registries have restrictions on publishing data based on small numbers. Numbers <3 were suppressed in Tuscany (Italy), numbers <5 were suppressed in Funen (Denmark) and in Wales; and numbers <8 were suppressed in the three English registries. The Northern Netherlands only released data if all exported results were rounded to the nearest five.

### Ethics

The EUROCAT registries follow national legislation as to whether parental consent is needed for registration of babies with anomalies and have ethical approval to conduct routine surveillance, data collection and transmission of anonymised data to the central database [11]. Registries obtained additional ethical approval to link their congenital anomaly data to administrative databases and submitted these approvals to the EUROlinkCAT ethics portfolio, as described in the study protocol [4]. The linked data files remained within the registries/institutions performing the linkage; only aggregate tables and analytic results were transferred to the Central Results Repository (CRR) at UU. UU obtained ethics permission to hold the CRR.

## Results

A total of 2,302,033 children born between 1995 and 2014 were extracted from the birth registers in national/vital statistics in the 11 participating regions, of which 2,199,379 were reference children and 102,654 were children with congenital anomalies as reported in the EUROCAT registry or in the Finnish congenital anomaly database (Fig 1). Table 2 shows that overall 97% of EUROCAT children with congenital anomalies were successfully matched to administrative databases with 10 of the 12 data sources (in 11 registries) linking >90% of children, and Tuscany and Zagreb matching 88% and 44% of children respectively. Due to the low linkage success, Zagreb was excluded from the remainder of the study. Overall, 95% of reference children were matched; out of eight data sources, six linked >94% of children, Emilia Romagna matched 85% and Valencian Region 75%.

**Table 2:** Percentage of children matched to hospital discharge or other administrative databases inside or outside of study period.

	EUROCAT children with congenital anomalies (%)	Reference children (%)
Denmark, Funen	>99.9	>99.9
Finland	99.4	99.7
Italy, Emilia Romagna	92.8	85.0
Italy, Tuscany	87.6	98.8
The Netherlands, North (LMR)	95.4	96.9
The Netherlands, North (LBZ)	94.9	94.8
Spain, Valencian Region	98.9	75.3
UK, Wales	98.9	99.8
UK, England, East Midlands & South Yorkshire	97.2	-
UK, England, Thames Valley	96.4	-
UK, England, Wessex	91.2	-
Croatia, Zagreb	43.8	-
Total ^a^	96.8	95.2

-No information on reference children

^a^ Excluding Zagreb, 97.3% of children with congenital anomalies were successfully matched

**Fig 2:**
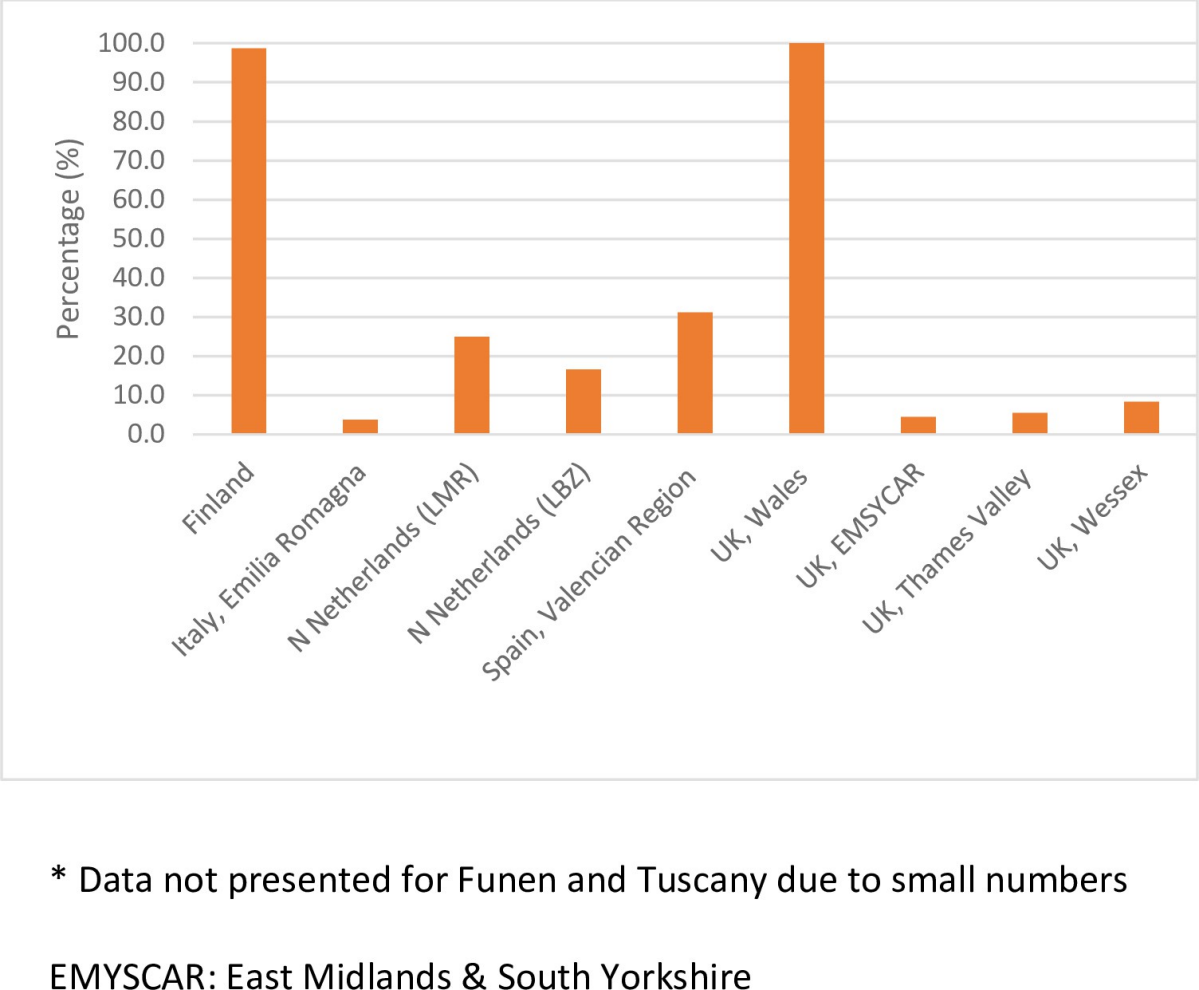
Proportion of non-matched children with congenital anomalies who died ≥1 day of age with no hospital admission recorded out of total children not matched to administrative database by registry*. * Data not presented for Funen and Tuscany due to small numbers EMYSCAR: East Midlands & South Yorkshire.

In total, 4.5% of all children were not matched to administrative databases. There was a 16-fold increase in the proportion of non-matched children with congenital anomalies who died ≥1 day of age (excluding Zagreb n = 610, 22.2%) compared to the non-matched reference children (n = 1,428, 1.4%), Fig 1. In Finland and Wales, where 1% of children with congenital anomalies were non-matched, almost all died ≥1 day of age (Fig 2). In the remaining regions, the proportion non-matched that died ≥1 day of age ranged from 4% in Emilia Romagna to 31% in Valencian Region. Neonatal deaths occurring in the first 28 days of life accounted for 61% of these deaths in Finland and 80% in Wales.

### Comparison of matched versus non-matched children

Compared to mothers aged 20–34 years, children with congenital anomalies born to teenage mothers (<20 years) and reference children born to mothers ≥35 years were less likely to be matched to hospital discharge or other databases after adjusting for regional variations (adjusted OR 0.59, 95% CI 0.53–0.66 and 0.86, 95% CI 0.85–0.87 respectively), (Table 3). The odds of being matched were higher for EUROCAT children born to mothers ≥35 years (adjusted OR 1.17, 95% CI 1.08–1.26) compared to those born to mothers aged 20–34 years. Overall, all children (i.e. reference children and those with congenital anomalies) born to teenage mothers and mothers ≥35 years were less likely to be matched compared with mothers aged 20–34 years (adjusted ORs 0.92, 95% CI 0.88–0.96; and 0.87, 95% CI 0.86–0.89 respectively).

**Table 3:** The unadjusted and adjusted Odds Ratio (OR) of being linked to any administrative databases according to characteristics of the mother and child reported in birth records for EUROCAT children and reference children ^a^.

		EUROCAT children		Reference children	
		Unadjusted OR	Adjusted OR^b^	Unadjusted OR	Adjusted OR^b^
Maternal age (years)	20–34	1.00	1.00	1.00	1.00
	<20	0.47 (0.42–0.52)	0.59 (0.53–0.66)	1.77 (1.69–1.86)	0.99 (0.94–1.04)
	≥35	1.01 (0.93–1.08)	1.17 (1.08–1.26)	0.50 (0.50–0.51)	0.86 (0.85–0.87)
Multiple birth	Singleton	1.00	1.00	1.00	1.00
	Twins or higher	0.90 (0.79–1.03)	0.77 (0.67–0.89)	0.92 (0.89–0.95)	0.99 (0.95–1.03)
Sex	Male	1.00	1.00	1.00	1.00
	Female	1.10 (1.04–1.17)	1.01 (0.95–1.07)	0.92 (0.91–0.93)	0.89 (0.88–0.90)
Gestational age (weeks)	≥37	1.00	1.00	1.00	1.00
	≤27	0.51 (0.40–0.64)	0.53 (0.42–0.67)	0.27 (0.25–0.30)	0.16 (0.14–0.17)
	28–31	0.52 (0.45–0.60)	0.52 (045–0.61)	0.80 (0.74–0.86)	0.76 (0.70–0.83)
	32–36	0.73 (0.67–0.79)	0.78 (0.71–0.85)	1.00 (0.97–1.03)	1.28 (1.24–1.32)
Birth weight (grams)	2,500–3,999	1.00	1.00	1.00	1.00
	<2,500	0.58 (0.54–0.63)	0.71 (0.65–0.77)	0.92 (0.90–0.95)	1.16 (1.13–1.20)
	≥4,000	1.49 (1.31–1.69)	1.08 (0.94–1.23)	2.85 (2.77–2.93)	1.19 (1.15–1.23)

^a^ Based on 9 registries (excluding Zagreb and Northern Netherlands)

^b^ Adjusted for region

Compared to singleton births, EUROCAT children who were twins/triplets were less likely to be matched to hospital or other databases (adjusted OR 0.77, 95% CI 0.67–0.89), although this association was not found for reference children, Table 3. Female reference children were less likely to be matched.

Preterm children born at ≤27 weeks gestational age were less likely to be matched compared to children born at term. The adjusted OR was 0.53 (95% CI 0.42–0.67) for EUROCAT children and 0.16 (95% CI 0.14–0.17) for reference children. Children born 28–31 weeks gestational age were also less likely to be matched compared to children born at term (adjusted OR 0.52, 95%CI 0.45–0.61 for EUROCAT children and 0.76, 95%CI 0.70–0.83 for reference children). Overall, all children (i.e. reference children and those with congenital anomalies) born at ≤27 weeks and 28–31 weeks were less likely to be matched (adjusted OR 0.23, 95% CI 0.21–0.25 and adjusted OR 0.75, 95% CI 0.70–0.81 respectively) compared with children born at term. Reference children born 32–36 weeks gestational age were more likely to be matched (adjusted OR 1.28, 95% CI 1.24–1.32). In contrast, EUROCAT children born 32–36 weeks gestational age were less likely to be matched (adjusted OR 0.78, 95% CI 0.71–0.85). The associations of low birthweight with probability of linkage were similar to those observed for low gestational ages for children with congenital anomalies, Table 3.

The OR of being matched to hospital discharge databases/found in other databases according to the characteristics in Table 3 are presented for each region individually and for all children combined (EUROCAT and reference children) (S2 Table). The pattern of associations were similar in all registries apart from Valencian Region where children born preterm, children born to teenage mothers, and multiple births were more likely to be matched to hospital discharge or other databases. In Wales, children born preterm, particularly those born ≤27 weeks, were less likely to be matched to administrative databases compared with children born at term.

### Completeness of matched data on potential risk factors in vital statistics (birth records)

Table 4 shows the level of complete information on potential risk factor variables for children with and without congenital anomalies in the linked datasets. Information on maternal age was known for at least 98% of children with congenital anomalies in all regions except for East Midlands & South Yorkshire where it was 92% and Wessex where it was 88%. It was known for >99% of reference children. Information on whether there was a singleton or multiple birth was known for 100% of children with congenital anomalies in all regions except East Midlands & South Yorkshire where it was 98% and was known for all reference children in all regions.

**Table 4:** Percentage of complete information on potential risk factors recorded in EUROCAT and vital statistics (birth records) for matched children, by region.

	Maternal age		Multiple birth		Sex		Gestational age at birth		Birthweight	
	CAs (%)	Ref (%)	CAs (%)	Ref (%)	CAs (%)	Ref (%)	CAs (%)	Ref (%)	CAs (%)	Ref (%)
Denmark, Funen	100	100	100	100	99.9	99.9	99.5	97.7	99.5	98.5
Finland	99.9	100	100	100	99.9	100	99.8	99.7	99.6	99.8
Italy, Emilia Romagna	98.6	99.9	100	100	99.9	99.9	98.8	99.9	99.4	99.9
Italy, Tuscany	99.1	99.9	100	100	99.9	100	99.7	99.9	99.5	99.8
The Netherlands, North (LMR)	98.6	-	100	-	100	-	93.3	-	92.9	-
The Netherlands, North (LBZ)	100	-	100	-	100	-	95.5	-	91.9	-
Spain, Valencian Region	97.7	99.4	100	100	100	100	97.8	97.5	96.9	97.4
UK, Wales	99.9	99.9	100	100	99.9	99.9	98.9	99.1	98.8	99.7
UK, E Midlands & S Yorkshire	91.60	-	97.6	-	99.9	-	93.1	-	91.6	-
UK, Thames Valley	99.3	-	100	-	99.7	-	98.9	-	99.5	-
UK, Wessex	88.4	-	100	-	99.8	-	99.8	-	40.8	-

-No information on reference children in the EUROlinkCAT study

CA = Children with congenital Anomalies; Ref = reference children

Information on sex was known for >99% of children with and without congenital anomalies in all regions. Gestational age at birth was known for >99% of children with congenital anomalies in 4 regions, for 95–99% in 5 regions and for 93% in 2 regions. It was known for >97% of reference children in all regions. Finally, birthweight was known for >90% of children with congenital anomalies in all regions except for Wessex where it was 41%, Table 4. It was known for >97% of reference children in all regions.

### Accuracy of information recorded in EUROCAT and in vital statistics (birth records)

A comparison of the accuracy of information recorded in EUROCAT and in vital statistics for children with congenital anomalies is presented in Table 5. For most registries, concordance between the linked datasets was >99% for infant sex and maternal age, and >95% for number of babies born and birthweight. There was greater discordance in recording gestational age with two registries having <80% agreement.

**Table 5:** Concordance between information recorded in EUROCAT registry and in vital statistics (birth records) for children with congenital anomalies.

	Maternal age	Multiple births	Infant sex	Gestational age	Birthweight
Denmark, Funen	99.8	99.5	99.7	89.5	98.0
Finland	99.9	99.9	100	98.9	99.7
Italy, Emilia Romagna	99.7	95.8	99.1	92.6	97.4
Italy, Tuscany	98.4	96.0	99.4	71.5	96.1
The Netherlands, North (LMR)	100	95.1	99.9	91.0	93.1
The Netherlands, North (LBZ)	100	96.3	100	94.2	94.0
Spain, Valencian Region	97.4	90.7	98.7	81.6	73.5
UK, Wales	98.7	99.6	99.4	80.5	97.7
UK, E Midlands & S Yorkshire	99.3	98.9	99.6	88.4	98.2
UK, Thames Valley	99.1	99.2	99.7	89.8	98.2
UK, Wessex	97.4	98.6	99.1	38.3	94.7

### Completeness of the linked data on key variables relating to hospital stay

The percentage of complete information on key variables relating to hospital admissions is shown in Table 6 for all children combined. Date of hospital admission and date of surgery were known for >99% of children in all regions. The date of discharge was >99% complete for all admissions in all regions except for Italy. The two Italian regions can have continuity of care whereby some children can have multiple stays in hospital but only the first admission and final discharge date are recorded.

**Table 6:** Percentage of matched children with complete information recorded on key variables relating to hospital stays.

	All hospital admissions ^a^				ICU admissions ^a^		
	Birth years included in study	Date of admission known (%)	Date of discharge known (%)	Date of surgery known (%)	Time period of ICU admissions	% of children with at least 1 ICU stay	% of children with ventilator use
Denmark, Funen	1995–2014	100	100	100	2005–2015^b^	13.0	1.8
Finland	1997–2014	100	>99	100	2010–2015^c^	10.8	1.2
Italy, Emilia Romagna	2008–2015	100	90^d^	100	2008–2015	6.6	2.0
Italy, Tuscany	2005–2015	100	82^d^	100	2005–2015	13.3	6.4
The Netherlands, North (LMR)	1995–2010	100	100	-	1995–2011 ^e^	-	-
The Netherlands, North (LBZ)	2013–2014	100	100	-	-	-	-
Spain, Valencian Region	2010–2014	100	100	100	2010–2015	3.8	1.8
UK, Wales	1998–2014	99.9	>99	100	-	-	1.0
UK, E Midlands & S Yorkshire ^f^	2003–2015	100	>99	>99	2003-Mar 2006	8.0	10.0^g^
UK, Thames Valley ^f^	2005–2013	100	>99	>99	2005-Mar 2006	14.0	8.0^g^
UK, Wessex ^f^	2004–2014	100	>99	100	2004-Mar 2006	7.0	5.0^g^

ICU = Intensive care unit. See S3 Table for codes used to identify ICU

^a^ Percentages calculated out of all children with a hospital stay

^b^ Information based on children born 2005–2014. ICU admission defined as child with a procedure code starting with NABA, NABB, NABC or NABE in any admission and/or child registered in neonatal department in first admission [“sengedage_neonatalafdeling_barn” (= bed-days in NICU child)]

^c^ Information on ICU admissions available from 2010–2015 for children born 2000–2014

^d^ Date of discharge was complete for all admissions, except for children with continuity of care involving multiple stays in hospital but only the first admission and final discharge date were recorded.

^e^ Data not included due to suboptimal recording of these data in hospital databases

^f^ Information on children with congenital anomalies only

^g^ Information on ventilator use available from 2006–2015

Information on ICU admissions was not available for the whole time period in Funen and Finland hence the proportions are calculated based on years with available data. The percentage of matched children with a stay in ICU ranged from 4% in Valencian Region to 14% in Thames Valley (Table 6).

Information on ventilator use was also not available for the whole time period in Funen, Finland, the 3 English registries and Wales, hence the proportions are calculated based on years with available data. The percentage of matched children with ventilator use ranged from 1% in Finland and Wales to 10% in East Midlands & South Yorkshire (Table 6).

## Discussion

This study reports on the accuracy of linking children with and without congenital anomalies to hospital databases in 11 European regions in 7 countries to assess if these data are suitable for use in EUROlinkCAT studies investigating morbidity outcomes in children. Overall, the linkage success was excellent as 97% of EUROCAT children and 95% of reference children were matched to administrative databases. Manual linkage is not appropriate due to the low linkage success rate.

Two regions, Emilia Romagna and Valencian Region had lower success in linking reference children than EUROCAT children. A possible explanation for the lower linkage success rate in Emilia Romagna is that probabilistic (e.g. date of birth) and deterministic linkage (e.g. unique ID number) were used to link EUROCAT children as the EUROCAT registry has details of each child registered with major congenital anomalies, whereas only deterministic linkage was used to link reference children. In addition, some hospitals in Emilia Romagna gave children a temporary unique ID that was not use for subsequent contacts with health providers hence these children could not be linked. In Valencian Region, the maximum age of follow-up is to a child’s 5^th^ birthday. This limits the amount of time children can be found in hospital discharge or prescription databases which may explain the higher rate of non-matched reference children in Valencian Region. The shorter follow-up period does not affect the matching rate for children with congenital anomalies in Valencian Region, as almost all children have a hospital stay in their first year of life [12]. It is also possible that some children may have been transferred outside the registry area for treatment. One registry, Zagreb, manually linked children with congenital anomalies which explained their lower linkage success rate.

In general, our comparison of matched and non-matched EUROCAT children showed that preterm births, children born to teenage mothers, and multiple births were less likely to be matched which is a potential source of bias. Preterm reference children were also less likely to be matched. The association between non-matched children and preterm births has been reported in an Australian study which found that non-matched children were more likely to be born preterm, to have low birthweight and to be in-hospital deaths [13]. In our study, we found the association with low birth weight for EUROCAT children but not for reference children, but our study categorised birthweights <2500 grams into a single group due to small numbers compared to the Australian study which classified birthweights into more granular categories i.e. <1000, 1000–1999 grams. The issue of being unable to match children born preterm may be overcome if linkage to a perinatal register is available.

Mortality for children with congenital anomalies is much higher than for children in the EU population [14, 15]. Our study showed that non-matched children with congenital anomalies were 16 times more likely to die ≥1 day of age compared to non-matched reference children, and that the majority of these deaths occurred in the neonatal period in Finland and Wales. An Australian validation study found that non-matched babies were more likely to have died on the day of birth [16]. Our results are not directly comparable to the Australian study as we excluded obstetric stays from our study as some countries record obstetric stays for the new-born, while other countries record the obstetric stay under the mother’s record.

Between 2–4% of reference children in six data sources had a congenital anomaly recorded in hospital discharge databases and hence were excluded as reference children in this study. Our research on the accuracy of congenital anomaly coding in hospital databases suggests that this is likely due to over-recording of congenital anomalies in hospital databases, rather -than under-reporting in EUROCAT [8]. Wales had the lowest proportion of congenital anomalies not recorded in EUROCAT which may be attributable to close working relationships between the registry and paediatric services, or due to the registry using major codes to classify minor congenital anomaly cases in EUROCAT. The highest proportion of congenital anomalies not recorded in EUROCAT was found in Tuscany, which is due to Tuscany using a 10% sample of reference children, rather than their whole population.

In this study, completeness of the linked data on potential risk factors was excellent, which suggests that these data are “fit for purpose” when it comes to analysing risk factors in relation to morbidity outcomes in this European paediatric population. There was one exception where birthweight was missing for half of children with congenital anomalies over the study period. In this instance, analyses using this variable were restricted to the years with good data i.e. the years where birthweight was recorded for at least 80% of cases [17]. Likewise, dates of hospital admission, discharge and surgeries are completed for >99% of children in the EUROlinkCAT linked datasets. However, while the completeness of the data are excellent, it must be recognised that the data recorded may not in fact be accurate, which could lead to misclassification bias. For example, there may be data entry errors relating to outcomes, risk factors or errors in the dates of hospital admission/discharge or in the recording of procedures, although we would expect these types of errors to be random. Overall, our results on the accuracy of information on children with congenital anomalies show a high concordance between information recorded in the EUROCAT registries and vital statistics/birth records, with some discordance found for gestational age in some regions.

We cannot assess the extent of inaccurate reporting or misclassification bias for reference children. However, a UK study compared demographic data recorded in neonatal electronic patient records to non-linked research data (the “gold” standard) and found low major discordance [18]. Discordance was 3% for gestational age, 0.9% for birthweight, 0.2% for sex, 1% for multiple birth status, and 1% for maternal year of birth. These findings suggest that demographic data tend to be well recorded in electronic records. The discordance was slightly higher for hospital interventions or outcomes e.g. major discordance was 4% for ICU admissions, 3% for length of hospital stay, 2% for month of discharge and 0.5% for year of discharge [18]. While these findings indicate that the data recorded in electronic patient records are of comparable quality to collected research data, a small degree of misclassification bias was found. The potential for inaccurate and/or missing data is a well-known limitation of data linkage studies as the data were not collected for research purposes. However, as hospital databases are often used to calculate payment for health care services, it is unlikely that a large proportion of hospital admissions were unreported. The study by Ford et al [16]. found that procedures were more likely to be accurately recorded than diagnoses.

The strength of this EUROlinkCAT linkage study is the use of the CDM that we developed to standardise the data, including potential risk factors, in different hospital databases across Europe. This can be used to assess morbidity in other populations as the variables such as date of hospital admission, age at admission etc. are the same regardless of the population under study. The CDM will allow future studies to be performed more efficiently [19]. A potential limitation is our decision to only link to hospital inpatient admissions, therefore outpatient visits are not evaluated in this study. Also, information on private hospitals may not be available for linkage. Finally, some children in Emilia Romagna and Valencian Region may be transferred outside their region or country for specialised treatment, and these admissions may not be available for linkage.

To conclude, this EUROlinkCAT study provides evidence of the linkage success in 11 European regions and the high quality of the linked data which indicate that these data are suitable for analysing morbidity outcomes in our paediatric population. The linkage was excellent in all but one region, which is a promising and important message for those conducting data linkage studies. Efforts are needed to improve linkages for children born preterm and those born to mothers aged <20 and ≥35 years. This study also provides information on the coding systems used in the hospital databases in 12 data sources contributing to this study which will be useful for future studies based on paediatric and other population groups.

## Supporting information

S1 Table EUROlinkCAT common data model (morbidity). (DOCX)

S2 Table Odds ratio of being included in the study by mother and baby characteristics, by region. (DOCX)

S3 Table Definition of intensive care in registries. (DOCX)
