# Supplementary material for: Creating a population-based cohort of children born with and without congenital anomalies using birth data matched to hospital discharge databases in 11 European regions: Assessment of linkage success and data quality
Source: PLoS One. 2023 Aug 30;18(8):e0290711. doi: 10.1371/journal.pone.0290711 (PMC10468043; doi:10.1371/journal.pone.0290711)
Supplement: S1 Table — (DOCX) [file pone.0290711.s001.docx]

S1 Table. EUROlinkCAT Common Data Model: Morbidity

Citation: Loane M, Densem J, de Walle H, Garne E, Given J, Karnell K, Pierini A, Rankin J, Reid A, Rissmann A, Tan J, Morris JK (2020). EUROlinkCAT Common Data Model: Morbidity. Ulster University. DOI: [10.13140/RG.2.2.29365.96484](http://dx.doi.org/10.13140/RG.2.2.29365.96484)

Contents

[**Introduction** 3](#_Toc72920126)

[**Table Overview** 4](#_Toc72920127)

[**Table structure** 5](#_Toc72920128)

[**Data Dictionary by Table** 6](#_Toc72920129)

[**Table: Patient level data** 6](#_Toc72920130)

[**Information on risk factors – needed for all children** 7](#_Toc72920131)

[**Recoded variables** 9](#_Toc72920132)

[**Derived variables relating to linkage** 10](#_Toc72920133)

[**Table: Hospital admission variables** 12](#_Toc72920134)

[**Table: Diagnosis variables (up to 10th birthday)** 13](#_Toc72920135)

[**Table: Surgery and Procedures (including admission to intensive care unit)** 15](#_Toc72920136)

[**Table: Prescriptions** 17](#_Toc72920137)

[**Appendix 1: Algorithm for classifying L_MORB_INCLUDE** 21](#_Toc72920138)

[**Appendix 2:** 22](#_Toc72920139)

NB: The common data model includes information on prescription variables which are not relevant to the current study

# **Introduction**

Sixteen EUROCAT congenital anomaly registries in nine countries across Europe participated in the EUROlinkCAT project assessing morbidity outcomes in children up to ten years of age born with a congenital anomaly. Each registry’s congenital anomaly data are already standardised according to EUROCAT’s definitions^[[1]](#footnote-1)^, but the local morbidity data are not. Registries obtain information on morbidity from their local hospital databases or their national/ vital statistics databases, which include data on births, hospital admissions/ discharges, diagnosis, surgery and prescriptions. The nine countries have different languages and healthcare systems. In addition, hospital databases in a registry area have their own structure, variable names, variable definitions, variable formats and coding schemes. EUROlinkCAT has developed a common data model (CDM) to standardise these data.

The CDM for standardising morbidity data consists of six tables as presented in the Table Overview i.e. there is a separate table for each of the following: patient data, EUROCAT congenital anomaly case data (already standardised1), hospital admissions, diagnoses, procedures and surgeries, and prescriptions. Unique identification numbers link the tables, as shown in Figure 1. Thus, irrespective of the structure of the original source data in each registry, the standardisation process creates a standardised dataset that provides the same structure and the same standardised variables across all participating registries. This ensures that data included in the EUROlinkCAT studies are comparable across registries thus facilitating distributed analysis.

# **Table Overview**

| **Table** | **Description** |
| --- | --- |
| Patient | There will be one row for each case and each control in this table. It will contain variables for which there is only one value per case or control (i.e. gender).  The data will be sourced from birth records, WP3 data, summarised hospital activity records and prescriptions. The data will contain information on both the child and its mother. |
| EUROCAT Case Data | This table holds the CA case information data. There will be one row for each of the cases in the patient table. Data is sourced from the EUROCAT file [file name: “XX_CA_cases_1995-2014v2] where XX represents the EUROCAT registry number |
| Hospital Admission | This table holds data relating to hospital admissions. There will be a row for each identifiable hospital admission, therefore each patient row may have zero, one or more hospital admission rows related to it.  Hospital stays relating to obstetric care, accident and emergency, and emergency room stays are excluded. |
| Diagnosis | This table holds information on diagnoses. There will be one row per diagnosis code and all diagnosis rows will be related to their specific hospital admission row. Each hospital admission may have zero, one or more diagnosis rows associated with it. |
| Procedures and surgeries | This table holds information on procedures/surgeries and admissions to Intensive Care Unit (ICU). There will be one row per procedures/surgeries code or ICU admission and all procedures and surgeries rows will be related to their specific hospital admission row. Each hospital admission may have zero, one or more procedures and surgeries rows associated with it. |
| Prescriptions | This table holds data on prescriptions for the following 5 conditions: Asthma, Cardiac, Diabetes, Epilepsy, and Infections.  Prescriptions for cases and controls will be derived from GP/ hospital outpatient pharmacy records.  There will be one row for each prescription. Each patient row may have zero, one or more prescription rows related to it. |

A visual representation of the table structure is presented below in Figure 1

# **Table structure**


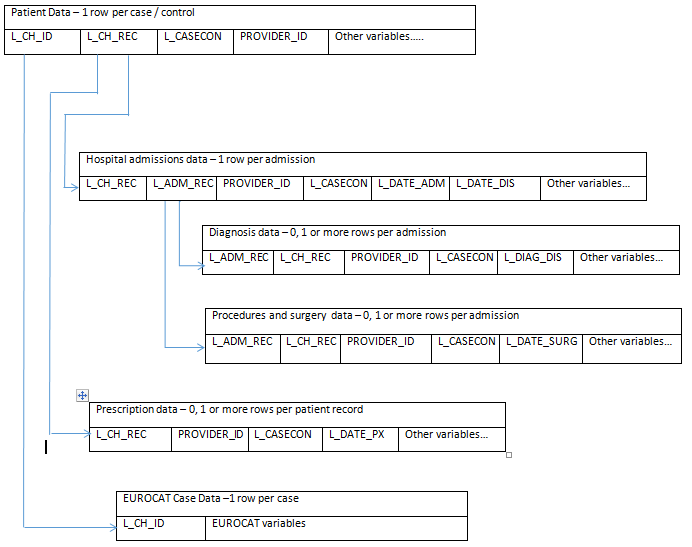


Figure 1: Table structure for EUROlinkCAT morbidity study

# **Data Dictionary by Table**

| ***Variable name*** | ***Variable definition/ description*** | ***Variable format*** | ***Variable values*** | ***Tables^[[2]](#footnote-2)^*** |
| --- | --- | --- | --- | --- |
| **Table: Patient level data** ***One row per child***  ***Core variables for linkage to hospital discharge records and for calculating age at event e.g. hospitalisation*** | | | | |
| L_CH_ID | Unique identifier of child  A unique ID that links child to another database | As recorded locally |  | - Patient  - EUROCAT case data |
| L_CH_REC | Unique child record number  Generated by syntax script for all children (cases and controls), irrespective of whether the child has been in hospital  For children with a hospital admission, this is used to link admission, diagnoses, procedures & surgeries and prescriptions to child | Numeric |  | - Patient  - Hospital admission  - Diagnoses  - Procedures & surgeries  - Prescription |
| PROVIDER_ID | Unique reference number as present in the provider’s linked data | String |  | - Patient  - Hospital admission  - Diagnoses  - Procedures & surgeries  - Prescription |
| L_CASECON | Case or control status (apply status AFTER linkage is complete)  Exclusions:  - child with gestational age <23 weeks  Control with ICD10 Q code or ICD9 (-CM) 740-759 codes recorded. This is a child with a congenital anomaly code (ICD10 Q code, or ICD9-CM 740-759) in hospital discharge records that is not recorded as a EUROCAT case.  Children coded as 1 or 2 will be included in WP4 analysis.  Children coded as 3 are required for WP6 | Numeric | 1= Case  2= Control with no ICD10 Q code or ICD9(-CM) 740-759 codes recorded  3=Control with ICD10 Q code or ICD9(-CM) 740-759 codes recorded | - Patient  - Hospital admission  - Diagnoses  - Procedures & surgeries  - Prescription |
| L_HOSP_ADM | Admitted to hospital (up to 10^th^ birthday)  Exclude obstetric stays i.e. hospital stays relating to the birth and Accident & Emergency/ Emergency room stays | Binary | 0= No hospital admissions  1= One or more hospital admission (s) | - Patient |
| L_PRESCRIPT | Prescription for one of the 5 specified conditions: Asthma, Cardiac, Diabetes, Epilepsy, and Infections.  N.B. UK prescriptions are issued by a GP, while in continental Europe, prescriptions are dispensed by the pharmacy (either community or hospital outpatient pharmacy) | Numeric | 0= No prescription  1= One or more prescription (s) | - Patient |
| L_CH_DATE_B | Child’s date of birth  Used to calculate age at hospitalisation, diagnoses, procedures, & prescriptions | DDMonYYYY |  | - Patient |
| L_CH_YEAR_B | Child’s year of birth | YYYY |  | - Patient |
| L_CH_DATE_D | Child’s date of death | DDMonYYYY |  | - Patient |
| L_CH_YEAR_D | Child’s year of death | YYYY |  | - Patient |
| L_CH_AGED_D | Age at death in complete days (up to 10th birthday).  A calculated field using the difference between date of death and death of birth i.e. subtract child’s date of birth from child’s date of death. | Numeric (1-4 digits) | 0 = died <24 hours after birth  1 = died 1 complete day after birth 2 = died 2 complete days after birth Etc. 8888 = Alive on 10th birthday or by end of study period, whichever is sooner  9999 = Died before 10th birthday, but exact time unknown | - Patient |
| **Information on risk factors – needed for all children** | | | |  |
| L_MATAGE_B | Maternal age at infant's birth in completed years | Numeric | 99 = Not known  . = Not recorded or not available for study | - Patient |
| L_MATMAR_STA | Maternal marital status at delivery | Numeric | 1 = Single  2 = Married/ Living together  3 = Widow  4 = Divorced/ Separated  9 = Not known  . = Not recorded or not available for study | - Patient |
| L_MAT_CTRY_B | Maternal country of birth/ place of birth/ country of origin | Numeric | 1= National  2= Other European  3 = Non-European  4= Non-national (exact nationality not specified)  9 = Not known  . = Not recorded or not available for study | - Patient |
| L_MAT_BMI | Maternal Body Mass Index (BMI) at first antenatal visit/at booking  Expected range 15 – 50 | Numeric  (Whole number only) | Exact BMI value  97 = <30  98 = >=30  99 = Not known  . = Not recorded or not available for study | - Patient |
| L_MAT_EDUC | Maternal education  (UNESCO’s International Standard Classification of Education (ISCED)) | Numeric | 1 = Pre-primary /Primary  2 = Any secondary  3 = Postsecondary (non tertiary)  4 = Tertiary  5=No education  9 = Not known  . = Not recorded or not available for study | - Patient |
| L_MATDEPR_IND | Deprivation index at maternal residence  Multiple deprivations scores are ranked into quintiles where 1= Least deprived and 5 = Most deprived (coding scheme used in Wales & Basque Country) | Numeric | 1 = First quintile (Least deprived)  2 = Second quintile  3 = Third quintile  4 = Fourth quintile  5 = Fifth quintile (Most deprived)  9 = Not known  . = Not recorded or not available for study | - Patient |
| L_PROXY_SES | Proxy variable for Socio-Economic Status (SES)  This is registry-specific. Use the agreed proxy variable for each registry.  Maternal education:   - Tertiary/ post-secondary=High - Any secondary = Middle - Primary/ pre-primary/ No education = Low   Maternal occupation:   - Professional = High - Intermediate= Middle - No occupation = Low   Multiple Deprivation Index   - Quintile 1 (Least deprived) =High - Quintiles 2-4= Middle - Quintile 5 (Most deprived) = Low | Numeric | 1 = High  2 = Middle  3 = Low  9=Not known  . = Not recorded or not available for study | - Patient |
| L_MULT_BIRTH | Singleton or multiple birth | Numeric | 1=Singleton  2=Twins  3=Triplets or higher  4= Multiple birth, number unknown  9= Not known  . = Not recorded or not available for study | - Patient |
| L_CH_SEX | Child’s sex | Numeric | 1 = Male  2 = Female  3 = Indeterminate  9 = Not known  . = Not recorded or not available for study | - Patient |
| L_CH_BW | Child’s birth weight (grams) | Numeric | 9999 = Not known  . = Not recorded or not available for study | - Patient |
| L_CH_GA_B | Child’s gestational age at birth (in completed weeks). | Numeric | 99 = Not known  . = Not recorded or not available for study | - Patient |
| L_PRENATAL | Prenatal diagnosis of congenital anomaly  [EUROCAT case only, i.e. L_CASECON=1  All control children in the linked dataset (i.e. L_CASECON=2 or L_CASECON=3) should be coded as .]  [Derived from the EUROCAT variables when discovered (WHENDISC) and age at prenatal diagnosis (AGEDISC)] | Numeric | 0 = Not prenatally diagnosed  1 = Prenatally diagnosed, <22 weeks  2 = Prenatally diagnosed, 22-31 weeks  3 = Prenatally diagnosed, 32+ weeks  4 = Prenatally diagnosed, GA unknown  9 = Not known if prenatally diagnosed  .= Not recorded or not available for study | -Patient |
| **Recoded variables** | | | | |
| Yeargp_WP4 | Grouped year of birth  1995/2004=1 2005/2009 =2  2010-2014 = 3 | Numeric | 1 = 1995-2004 2 = 2005-2009  3 = 2010 - 2014 | -Patient |
| Yeargp_Px | Grouped year of birth for the prescription study  2000/2004=1 2005/2009 =2  2010-2014 = 3 | Numeric | 1 = 2000-2004 2 = 2005-2009  3 = 2010 - 2014 | -Patient |
| BMI_gp | Grouped BMI  BMI <10, code as unknown  BMI >60, code as unknown  Blank or missing, code as unknown | Numeric | 1 = <30  2 = 30+  9 = unknown | -Patient |
| BW_WP4_gp | Grouped Birth weight (BW)  BW <400g, code as unknown  BW >7000g, code as unknown  Blank or missing, code as unknown | Numeric | 1= <1500g  2 = 1500-2499g  3 = 2500-3999g  4 = 4000+ g  9 = unknown | -Patient |
| GA_WP4_gp | Grouped Gestational age  GA <23 weeks, excluded from study  GA >44 weeks, code as unknown  Blank or missing, code as unknown | Numeric | 1 = 23-27 weeks  2 = 28-31 weeks  3 = 32-36 weeks  4 = 37+ weeks  9 = unknown | -Patient |
| GA_disc_gp | Grouped Gestational age at discovery  GA at discovery <8 weeks, code as unknown  GA at discovery >42 weeks, code as unknown  Blank or missing, code as unknown | Numeric | 1= <22 weeks  2 = 22-31 weeks  3 = 32+ weeks  9 = unknown | -Patient |
| Matage_gp | Grouped Maternal age at infant’s birth  Maternal age range  10-19 years, code =1  20-34 years, code=2  35-59 years, code=3  All other values, blanks or missing, code=9 | Numeric | 1= <20 years  2= 20-34 years  3= 35+ years  9=Not known | -Patient |
| **Derived variables relating to linkage** | | | | |
| L_MATCH_TYPE_V | Match with national/ vital statistics database | Numeric | 1 = Linkage to national/vital statistics database - match  2 = Linkage to national/vital statistics database - non-match  3 = EUROCAT death only  . = No linkage to national/ vital statistics | -Patient |
| L_MATCH_TYPE_H | Match with hospital database  The value 4 “Only matched to hospital discharge database outside the study period or matched to hospital outpatient records in the study period” is included for matching purposes only i.e. the hospital stay for this child is not included in analysis.  The rationale is that if a child is not matched to hospital discharge records during the study period, but is found in hospital records AFTER the study period, then we can be more confident that the child did not have a hospital admission during our study period.  Similarly, if a child is found in outpatient records during the study period but is not found in the hospital discharge records, we can be more confident that the child did not have a hospital admission during our study period. | Numeric | 1 = Linkage to hospital database - match  2 = Linkage to hospital database - non-match  3 = EUROCAT death only  4 = Only matched to hospital discharge database outside the study period or matched to hospital outpatient records in the study period | -Patient |
| L_CONFID_HDR | Strength of match with hospital database.  Use local data provider’s codes for assessing confidence that the case is correctly matched. If local code unavailable, use suggested coding  The value 0 represents a child:   - not matched to hospital discharge records in the study period, but found in other databases within national statistics (such as outpatient records) - found only in hospital discharge records AFTER the study period. | Numeric | 0=Found in other database or found only in hospital database outside the study period  1=Excellent  2=Good  3=Fair  4=Poor  9=Not Matched | - Patient |
| L_MORB_INCLUDE | Include child in WP4 analysis  Used in sensitivity analysis to assess if a child should be included in the WP4 analysis or not i.e. it will assess confidence that a child definitely did not go to hospital if hospital admission (variable L_HOSP_ADM = 0).  Please see appendix 1 for algorithm) | Binary | 0 = Do not include in analysis  1 = Include in analysis | - Patient |
| L_MATCH_PX | Match with GP / community pharmacy prescription database / hospital outpatient pharmacy records | Numeric | 1 = Linked prescription database – match  2 = Linked to prescription database – non-match  3 = Linkage to prescription data not available | - Patient |
| L_CONFID_PX | Strength of match with prescription database.  The value 9 represents a child not matched/ found in the local GP/ pharmacy prescription records (i.e. L_MATCH_PX=2);  or a child whose GP/ pharmacy do not contribute data to this study (i.e. L_MATCH_PX=3).  Children coded as L_MATCH_PX=3 will be excluded from analysis. | Numeric | 1=Excellent  2=Good  3=Fair  4=Poor  9=Not Matched | - Patient |
| L_DATE_LOST | Date lost to follow-up/ linkage (i.e. due to emigration, adoption or other reason) | DDMonYYYY | . = Not recorded or not available for study | - Patient |
| L_YEAR_LOST | Year lost to follow-up/ linkage (i.e. due to emigration, adoption or other reason) | YYYY | . = Not recorded or not available for study | - Patient |
| L_AGEL_D | Age lost to follow-up/ linkage in complete days | Numeric (1-4 digits) | . = Not recorded or not available for study | - Patient |
| L_EXIT_DATE | Date of last day in study (censored/lost/died/alive)  - Date lost - if child was lost to follow up  - Date of death - if child has died  - Date of birth plus 3652 days (approx. 10th birthday) if child was born on or before the 31st Dec 2005 and:  (i) is definitively known to be alive on 10th birthday; or (ii) there is no information on death or lost to follow-up  - Date of last day of the study (31st Dec 2015) if child was born on or after 1st Jan 2006 and:  (i) is definitively known to be alive on 31st Dec 2015; or (ii) there is no information on death or lost to follow-up | DDMonYYYY |  | - Patient |
| L_EXIT_DAYS | Number of days child is in study.  This is calculated as the last date child was in the study (L_EXIT_DATE) minus the child’s birth date (L_CH_DATE_B). | Numeric (1-4 digits) |  | - Patient |
| L_CH_STATUS | Outcome status  -Died = child is known to have died before 10th birthday or 31 Dec 2015 (whichever earlier)  -Alive at 10^th^ birthday = child was born on or before the 31st Dec 2005 and:  (i) is definitively known to be alive on 10th birthday; or (ii) there is no information on death or lost to follow-up  - Censored on 31^st^ Dec 2015 = child was born on or after 1st Jan 2006 and:  (i) is definitively known to be alive on 31st Dec 2015; or (ii) there is no information on death or lost to follow-up  -Lost to follow up = child is lost to follow-up/ linkage (i.e. due to emigration, adoption or other reason) | Numeric | 1 = Died  2 = Alive at 10^th^ birthday  3 = Censored on 31^st^ Dec 2015  4 = Lost to follow up | - Patient |
| **Table: Hospital admission variables**  ***(excluding Obstetric stays and Accident & Emergency/ Emergency room stays)***  ***One row for each hospital admission.***  ***If multiple hospital admissions, please complete all hospital admission variables for each admission*** | | | | |
| L_CH_REC | Unique child record number  Generated by syntax script for all children (cases and controls), irrespective of whether the child has been in hospital  For children with a hospital admission, this is used to link admission, diagnoses, procedures & surgeries and prescriptions to child | Numeric |  | - Patient  - Hospital admission  - Diagnoses  - Procedures & surgeries  - Prescription |
| PROVIDER_ID | Unique reference number as present in the provider’s linked data | String |  | - Patient  - Hospital admission  - Diagnoses  - Procedures & surgeries  - Prescription |
| L_ADM_REC | Unique admission record number  Used to link the child’s admission records to the child’s diagnoses and procedures & surgeries during that admission  Generated by syntax script | Numeric |  | - Hospital admission - Diagnoses  - Procedures & surgeries |
| L_CASECON | Case or control status (apply status AFTER linkage is complete)  Exclusions:  - child with gestational age <23 weeks  Control with ICD10 Q code or ICD9 (-CM) 740-759 codes recorded. This is a child with a congenital anomaly code (ICD10 Q code, or ICD9-CM 740-759) in hospital discharge records that is not recorded as a EUROCAT case.  Children coded as 1 or 2 will be included in WP4 analysis.  Children coded as 3 are required for WP6 | Numeric | 1= Case  2= Control with no ICD10 Q code or ICD9(-CM) 740-759 codes recorded  3=Control with ICD10 Q code or ICD9(-CM) 740-759 codes recorded | - Patient  - Hospital admission  - Diagnoses  - Procedures & surgeries  - Prescription |
| L_HOSP_SEQ | **This variable is only required for the registries: registry numbers 13, 28**  Sequential number allocated to each hospital in registry area e.g. Hospital A=1, Hospital B=2 etc.  Purpose of this variable is to identify the hospital for each record. It is known that some hospitals in some registries are missing >20% hospital admissions in specific years. This variable will be used in sensitivity analysis, to include/ exclude the hospital (s) with >20% missing information to see its effect on the analysis. | Numeric | . = Not recorded or not available for study | - Hospital admission |
| L_DATE_ADM | Date of admission to hospital | DDMonYYYY |  | - Hospital admission |
| L_YEAR_ADM | Year of admission to hospital | YYYY |  | - Hospital admission |
| L_DATE_DIS | Date of discharge from hospital | DDMonYYYY |  | - Hospital admission |
| L_YEAR_DIS | Year of discharge from hospital | YYYY |  | - Hospital admission |
| L_HOSP_DAYS | Length of stay in hospital in days  Subtract discharge date from hospital admission date.  If value =0 for children admitted and discharged on the same day, replace value 0 with value 0.5 | Numeric | 0.5 = <1 day  1=1 day/ over-night stay  2=2 days etc  999= Not known | - Hospital admission |
| L_AGE_ADM | Child’s age at hospital admission in days (up to 10^th^ birthday).  Subtract child’s date of birth from date of admission to hospital | Numeric | 0= <1 day old  1= 1 day old  2= 2 days old etc  9999= exact age not known | - Hospital admission |
| L_ICU_ADM | Admission to Neonatal, Paediatric or other Intensive Care Unit (ICU) during this hospital admission | Numeric | 0= No ICU admission  1=ICU admission  9 = Not known  . = Not recorded or not available for study | - Hospital admission |
| L_VENT | Mechanical ventilator use during this hospital admission  Non-invasive ventilation is excluded | Numeric | 0= No  1= Yes  9= Not known  . = Not recorded or not available for study | - Hospital admission |
| **Table: Diagnosis variables (up to 10th birthday)**  ***Each diagnosis is a separate observation (row) in the table***  ***0, 1 or more rows for each hospital admission***  ***If multiple diagnoses, please provide date of each diagnosis*** | | | | |
| L_CH_REC | Unique child record number  Generated by syntax script for all children (cases and controls), irrespective of whether the child has been in hospital  For children with a hospital admission, this is used to link admission, diagnoses, procedures & surgeries and prescriptions to child | Numeric |  | - Patient  - Hospital admission  - Diagnoses  - Procedures & surgeries  - Prescription |
| PROVIDER_ID | Unique reference number as present in the provider’s linked data | String |  | - Patient  - Hospital admission  - Diagnoses  - Procedures & surgeries  - Prescription |
| L_ADM_REC | Unique admission record number  Used to link the child’s admission records to the child’s diagnoses and procedures during that admission  Generated by syntax script | Numeric |  | - Hospital admission  - Diagnoses  - Procedures & surgeries |
| L_CASECON | Case or control status (apply status AFTER linkage is complete)  Exclusions:  - child with gestational age <23 weeks  Control with ICD10 Q code or ICD9 (-CM) 740-759 codes recorded. This is a child with a congenital anomaly code (ICD10 Q code, or ICD9-CM 740-759) in hospital discharge records that is not recorded as a EUROCAT case.  Children coded as 1 or 2 will be included in WP4 analysis.  Children coded as 3 are required for WP6 | Numeric | 1= Case  2= Control with no ICD10 Q code or ICD9(-CM) 740-759 codes recorded  3=Control with ICD10 Q code or ICD9(-CM) 740-759 codes recorded | - Patient  - Hospital admission  - Diagnoses  - Procedures & surgeries  - Prescription |
| L_DIAG | Diagnosis in ICD9 or ICD10 for the hospital stay | String  As recorded in the hospital database |  | - Diagnoses |
| L_DATE_DIAG | Date of diagnosis  Use date of discharge as a proxy for date of diagnosis, if date of diagnosis is not available | DDMonYYYY |  | - Diagnoses |
| L_AGE_DIAG | Child’s age at diagnosis in days (up to 10^th^ birthday).  Subtract child’s date of birth from date of diagnosis to calculate the child’s age at diagnosis. If date of diagnosis is not available, then use date of discharge | Numeric | 0= <1 day old  1= 1 day old  2= 2 days old etc  9999= exact age not known | - Diagnoses |
| L_CANCER | A diagnosis of cancer (ICD9 or ICD10) | Binary | 0 = No  1 = Yes | - Diagnoses |
| L_CPALSY | A diagnosis of cerebral palsy (ICD9 or ICD10) | Binary | 0 = No  1 = Yes | - Diagnoses |
| L_EPILEPSY | A diagnosis of epilepsy or seizures (ICD9 or ICD10) | Binary | 0 = No  1 = Yes | - Diagnoses |
| L_RENAL | A diagnosis of renal failure (ICD9 or ICD10) | Binary | 0 = No  1 = Yes | - Diagnoses |
| L_HEARING | A diagnosis of hearing loss (ICD9 or ICD10) | Binary | 0 = No  1 = Yes | - Diagnoses |
| L_VISION | A diagnosis of visual impairment or blindness (ICD9 or ICD10) | Binary | 0 = No  1 = Yes | - Diagnoses |
| L_INJURYPOISON | A diagnosis of injury or poisoning (ICD9 or ICD10) | Binary | 0 = No  1 = Yes | - Diagnoses |
| L_BATTERED | A diagnosis of physical abuse (battered child) (ICD9 or ICD10) | Binary | 0 = No  1 = Yes | - Diagnoses |
| **Table: Surgery and Procedures (including admission to intensive care unit)** ***Each surgery/ procedure /ICU admission is a separate observation (row) in the table***  ***0,1 or more rows for each hospital admission***  ***If multiple procedures, please provide procedure date and age of child at each procedure*** | | | | |
| L_CH_REC | Unique child record number  Generated by syntax script for all children (cases and controls), irrespective of whether the child has been in hospital  For children with a hospital admission, this is used to link admission, diagnoses, procedures & surgeries and prescriptions to child | Numeric |  | - Patient  - Hospital admission  - Diagnoses  - Procedures & surgeries  - Prescription |
| PROVIDER_ID | Unique reference number as present in the provider’s linked data | String |  | - Patient  - Hospital admission  - Diagnoses  - Procedures & surgeries  - Prescription |
| L_ADM_REC | Unique admission record number  Used to link the child’s admission records to the child’s diagnoses and procedures during that admission  Generated by syntax script |  |  | - Hospital admission  - Diagnoses  - Procedures & surgeries |
| L_CASECON | Case or control status (apply status AFTER linkage is complete)  Exclusions:  - child with gestational age <23 weeks  Control with ICD10 Q code or ICD9 (-CM) 740-759 codes recorded. This is a child with a congenital anomaly code (ICD10 Q code, or ICD9-CM 740-759) in hospital discharge records that is not recorded as a EUROCAT case.  Children coded as 1 or 2 will be included in WP4 analysis.  Children coded as 3 are required for WP6 | Numeric | 1= Case  2= Control with no ICD10 Q code or ICD9(-CM) 740-759 codes recorded  3=Control with ICD10 Q code or ICD9(-CM) 740-759 codes recorded | - Patient  - Hospital admission  - Diagnoses  - Procedures & surgeries  - Prescription |
| L_SURG_DATE | Date of surgery / procedure  If date of surgery is missing, use the date of ICU admission as a proxy. If date of ICU is also missing, use date of discharge | DDMonYYYY |  | - Procedures & surgeries |
| L_SURG_YEAR | Year of surgery / procedure | YYYY |  | - Procedures & surgeries |
| L_SURG_AGE | Child’s age at surgery in days (up to 10^th^ birthday).  Subtract child’s date of birth from date of surgery / procedure | Numeric | 0= <1 day old  1= 1 day old  2= 2 days old etc  9999=surgery, but exact age not known | - Procedures & surgeries |
| L_SURG_CODE | Code for surgery / procedure performed during this hospital stay  As recorded in the hospital database | String |  | - Procedures & surgeries |
| SU_ANY | Any Surgery  As recorded in hospital database | Binary | 0 = No  1 = Yes | - Procedures & surgeries |
| SU_FEEDING | Surgical Feeding Tube inserted  As recorded in hospital database | Binary | 0 = No  1 = Yes | - Procedures & surgeries |
| SU_SB | Surgery for spina bifida  As recorded in hospital database | Binary | 0 = No  1 = Yes | - Procedures & surgeries |
| SU_HYDROSHUNT | Revisions of shunt surgery – relevant to both spina bifida and hydrocephaly  As recorded in hospital database | Binary | 0 = No  1 = Yes | - Procedures & surgeries |
| SU_EYE | Eye surgery  As recorded in hospital database | Binary | 0 = No  1 = Yes | - Procedures & surgeries |
| SU_CARDIAC | Any Cardiac surgery  As recorded in hospital database | Binary | 0 = No  1 = Yes | - Procedures & surgeries |
| SU_RASHKIND | Rashkind procedure  As recorded in hospital database | Binary | 0 = No  1 = Yes | - Procedures & surgeries |
| SU_TGA_ARTERIAL | Arterial switch  As recorded in the hospital database | Binary | 0 = No  1 = Yes | - Procedures & surgeries |
| SU_TGA_ATRIAL | Atrial switch  As recorded in the hospital database | Binary | 0 = No  1 = Yes | - Procedures & surgeries |
| SU_VSD | Surgery for ventricular septal defect  As recorded in hospital database | Binary | 0 = No  1 = Yes | - Procedures & surgeries |
| SU_ASD | Surgery for atrial septal defect  As recorded in hospital database | Binary | 0 = No  1 = Yes | - Procedures & surgeries |
| SU_AVSD | Surgery for atrioventricular septal defect  As recorded in hospital database | Binary | 0 = No  1 = Yes | - Procedures & surgeries |
| SU_COARCT | Surgery for coarctation of aorta  As recorded in hospital database | Binary | 0 = No  1 = Yes | - Procedures & surgeries |
| SU_PDA | Surgery for Patent ductus arteriosus (PDA) or catheter closure  As recorded in hospital database | Binary | 0 = No  1 = Yes | - Procedures & surgeries |
| SU_BLALOCK | Blalock shunt  As recorded in hospital database | Binary | 0 = No  1 = Yes | - Procedures & surgeries |
| SU_HEMIFON | Hemi-Fontan Surgery  As recorded in hospital database | Binary | 0 = No  1 = Yes | - Procedures & surgeries |
| SU_FONTAN | Complete Fontan surgery  As recorded in hospital database | Binary | 0 = No  1 = Yes | - Procedures & surgeries |
| SU_CLEFT | Cleft surgery  As recorded in hospital database | Binary | 0 = No  1 = Yes | - Procedures & surgeries |
| SU_OESOPH | Surgery for oesophageal atresia  As recorded in hospital database | Binary | 0 = No  1 = Yes | - Procedures & surgeries |
| SU_DILATION | Oesophageal atresia dilation  As recorded in hospital database | Binary | 0 = No  1 = Yes | - Procedures & surgeries |
| SU_INTESTINE | Intestinal surgery  As recorded in hospital database | Binary | 0 = No  1 = Yes | - Procedures & surgeries |
| SU_STOMY | Stoma Surgery  As recorded in the hospital database | Binary | 0 = No  1 = Yes | - Procedures & surgeries |
| SU_KASAI | Liver/bile surgery (Kasai)  As recorded in hospital database | Binary | 0 = No  1 = Yes | - Procedures & surgeries |
| SU_CDH | Surgery for diaphragmatic hernia  As recorded in hospital database | Binary | 0 = No  1 = Yes | - Procedures & surgeries |
| SU_GASTRO | Surgery for gastroschisis  As recorded in hospital database | Binary | 0 = No  1 = Yes | - Procedures & surgeries |
| SU_RENAL | Renal surgery  As recorded in hospital database | Binary | 0 = No  1 = Yes | - Procedures & surgeries |
| SU_HYPOSPA | Surgery for hypospadias  As recorded in hospital database | Binary | 0 = No  1 = Yes | - Procedures & surgeries |
| SU_TRANY | Transplantation (heart, kidney or liver)  As recorded in hospital database | Binary | 0 = No  1 = Yes | - Procedures & surgeries |
| SU_TRHEART | Transplantation (heart)  As recorded in hospital database | Binary | 0 = No  1 = Yes | - Procedures & surgeries |
| SU_TRKIDNEY | Transplantation (kidney)  As recorded in hospital database | Binary | 0 = No  1 = Yes | - Procedures & surgeries |
| SU_TRLIVER | Transplantation (liver)  As recorded in hospital database | Binary | 0 = No  1 = Yes | - Procedures & surgeries |
| L_ICU_DATE_ADM | Date of admission to ICU | DDMonYYYY | . = Not recorded or not available for study | - Procedures & surgeries |
| L_ICU_DATE_DIS | Date moved from ICU to other ward | DDMonYYYY | . = Not recorded or not available for study | - Procedures & surgeries |
| L_ICU_YEAR | Year of admission to ICU | YYYY | . = Not recorded or not available for study | - Procedures & surgeries |
| L_ICU_AGE | Child’s age at admission to ICU in days (up to 10^th^ birthday).  Subtract child’s date of birth from date of ICU admission | Numeric | 0= <1 day old  1= 1 day old  2= 2 days old etc  9999= exact age not known  . = Not recorded by registry or not available for study | - Procedures & surgeries |
| **Table: Prescriptions**  ***(for 5 conditions only: Asthma, Cardiac, Diabetes, Epilepsy and Infections)***  ***One row for each prescription.***  ***If multiple prescriptions, please provide prescription date and age of child at each prescription*** | | | | |
| L_CH_REC | Unique child record number  Generated by syntax script for all children (cases and controls), irrespective of whether the child has been in hospital  For children with a hospital admission, this is used to link admission, diagnoses, procedures & surgeries and prescriptions to child | Numeric |  | - Patient  - Hospital admission  - Diagnoses  - Procedures & surgeries  - Prescriptions |
| PROVIDER_ID | Unique reference number as present in the provider’s linked data | String |  | - Patient  - Hospital admission  - Diagnoses  - Procedures & surgeries  - Prescription |
| L_CASECON | Case or control status (apply status AFTER linkage is complete)  Exclusions:  - child with gestational age <23 weeks  Control with ICD10 Q code or ICD9 (-CM) 740-759 codes recorded. This is a child with a congenital anomaly code (ICD10 Q code, or ICD9-CM 740-759) in hospital discharge records that is not recorded as a EUROCAT case.  Children coded as 1 or 2 will be included in WP4 analysis.  Children coded as 3 are required for WP6 | Numeric | 1= Case  2= Control with no ICD10 Q code or ICD9(-CM) 740-759 codes recorded  3=Control with ICD10 Q code or ICD9(-CM) 740-759 codes recorded | - Patient  - Hospital admission  - Diagnoses  - Procedures & surgeries  - Prescription |
| L_AGE_PX | Child’s age at prescription in complete days (up to 10^th^ birthday).  Subtract child’s date of birth from the date prescription was issued/ dispensed | Numeric | 0= <1 day old  1= 1 day old  2= 2 days old etc  9999= exact age not known  . = Not recorded or not available for study | - Prescriptions |
| L_DATE_PX | Date prescription was issued/ dispensed | DDMonYYYY | . = Not recorded or not available for study | - Prescriptions |
| L_DRUG_CODE | Codes for medication: Anatomical Therapeutic Chemical classification (ATC), or Read codes | As recorded in local prescription database |  | - Prescriptions |
| DIA_ANY | Any prescribed antidiabetic medication  (ATC codes beginning A10) | Binary | 0 = No  1 = Yes | - Prescriptions |
| DIA_INS | Any prescribed Insulin or insulin analogues  (ATC codes beginning A10A) | Binary | 0 = No  1 = Yes | - Prescriptions |
| DIA_GLU_LOW | Any prescribed blood glucose lowering drugs, excluding insulins  (ATC codes beginning A10B) | Binary | 0 = No  1 = Yes | - Prescriptions |
| INF_ANY | Any prescribed anti-infective medication  (ATC codes beginning J01-J05) | Binary | 0 = No  1 = Yes | - Prescriptions |
| INF_ANTIBAC_SYS | Any prescribed antibacterials for systemic use  (ATC codes beginning J01) | Binary | 0 = No  1 = Yes | - Prescriptions |
| INF_PEN | Any prescribed beta-lactam antibacterials, penicillins  (ATC codes beginning J01C) | Binary | 0 = No  1 = Yes | - Prescriptions |
| INF_MAC | Any prescribed macrolides, lincosamides and streptogramins  (ATC codes beginning J01F) | Binary | 0 = No  1 = Yes | - Prescriptions |
| INF_ANTIVIR_SYS | Any prescribed antivirals for systemic use  (ATC codes beginning J05) | Binary | 0 = No  1 = Yes | - Prescriptions |
| EPI_ANY | Any prescribed antiepileptic drugs (AEDs)  (ATC codes beginning N03) | Binary | 0 = No  1 = Yes | - Prescriptions |
| EPI_OLD | Any prescribed older AEDs  (ATC codes beginning N03AA phenobarbital, N03AB phenytoin, N03AE clonazepam, N03AF carbamazepine) | Binary | 0 = No  1 = Yes | - Prescriptions |
| EPI_VPA | Any prescribed fatty acid derivatives  (ATC codes beginning N03AG, valproic acid) | Binary | 0 = No  1 = Yes | - Prescriptions |
| EPI_NEW | Any prescribed newer AEDs  (ATC codes N03AX lamotrigine, topiramate, gabapentin, levetiracetam) | Binary | 0 = No  1 = Yes | - Prescriptions |
| AST_ANY | Any prescribed antiasthmatic medications  (ATC codes beginning R03) | Binary | 0 = No  1 = Yes | - Prescriptions |
| AST_IN_B2 | Any prescribed Inhaled β2- agonists  (ATC codes beginning R03AC) | Binary | 0 = No  1 = Yes | - Prescriptions |
| AST_IN_CORT | Any prescribed Inhaled corticosteroids  (ATC codes beginning R03BA) | Binary | 0 = No  1 = Yes | - Prescriptions |
| AST_ANTICHO | Any prescribed Anticholinergic inhaled medications  (ATC codes beginning R03BB) | Binary | 0 = No  1 = Yes | - Prescriptions |
| AST_ANTIALL | Any prescribed Antiallergic agents, excl. corticosteroids  (ATC codes beginning R03BC) | Binary | 0 = No  1 = Yes | - Prescriptions |
| AST_LEU | Any prescribed Leukotriene receptor antagonists  (ATC codes beginning R03DC) | Binary | 0 = No  1 = Yes | - Prescriptions |
| CAR_ANY | Any prescribed cardiac medication, excluding quinidine and adrenalin pen  (ATC codes beginning C01-C03, C07-C09, excluding C01BA51, C01BA71, C01CA24) | Binary | 0 = No  1 = Yes | - Prescriptions |
| CAR_VWC1 | Any prescribed sodium channel blockers Vaughan Williams class (VWC) 1  (ATC codes beginning C01BA, C01BB and C01BC, excluding C01BA51, C01BA71 quinidine) | Binary | 0 = No  1 = Yes | - Prescriptions |
| CAR_VWC2 | Any prescribed betablockers VWC 2  (ATC codes beginning C07A) | Binary | 0 = No  1 = Yes | - Prescriptions |
| CAR_VWC3 | Any prescribed VWC 3 medications (amiodarone)  (ATC codes beginning C01BD) | Binary | 0 = No  1 = Yes | - Prescriptions |
| CAR_VWC5 | Any prescribed VWC 5 medications (digoxine)  (ATC codes beginning C01AA05) | Binary | 0 = No  1 = Yes | - Prescriptions |
| CAR_HYP | Any prescribed medications for hypertension (calcium antagonists and agents acting on renin-angiotensin system)  (ATC codes beginning C08, C09) | Binary | 0 = No  1 = Yes | - Prescriptions |
| CAR_DIU | Any prescribed diuretics  (ATC codes beginning C03) | Binary | 0 = No  1 = Yes | - Prescriptions |
| L_GP_HOSP | Indicates if community pharmacy or hospital outpatient pharmacy prescription (for Italian registries only) | Numeric | 1= Community pharmacy prescription  2= Hospital outpatient pharmacy prescription  3= Not available (for non-Italian registries) | - Prescriptions |

## **Appendix 1: Algorithm for classifying L_MORB_INCLUDE**

If L_MATCH_TYPE_V=1 & L_MATCH_TYPE_H=1 & L_CONFID_HDR =1 or 2 or 3, then L_MORB_INCLUDE **=1** [child matched to national statistics, and matched to hospital records – confidence is excellent, good, fair, so we are confident that this child attended hospital].

If L_MATCH_TYPE_V=1 & L_MATCH_TYPE_H=1 & L_CONFID_HDR =4, then L_MORB_INCLUDE =0 [child matched to national statistics, and child matched to hospital records – confidence is poor], therefore not confident that the child is correctly matched to the right hospital record]

If L_MATCH_TYPE_V=1 & L_MATCH_TYPE_H=2 & L_CONFID_HDR =0, then L_MORB_INCLUDE =1 [child matched to national statistics, not matched to hospital records, but found in other databases, so reasonably confident that the child did not attend hospital]

If L_MATCH_TYPE_V=1 & L_MATCH_TYPE_H=2 & L_CONFID_HDR =9, then L_MORB_INCLUDE =0 [child matched to national statistics, but not matched to hospital records, so we don’t know if this child attended hospital or not]

If L_MATCH_TYPE_V=2 & L_MATCH_TYPE_H=1 & L_CONFID_HDR =1, 2, or 3, then L_MORB_INCLUDE =1 [child not matched to national statistics, matched to hospital records, confidence is excellent, good, fair], so we are confident that the child attended hospital

If L_MATCH_TYPE_V=2 & L_MATCH_TYPE_H=1 & L_CONFID_HDR =4, then L_MORB_INCLUDE =0 [child not matched to national statistics, but matched to hospital records, confidence is poor, so not confident that the child is correctly matched to the right hospital record]

If L_MATCH_TYPE_V=2 & L_MATCH_TYPE_H=2 & L_CONFID_HDR =9, then L_MORB_INCLUDE =0 [child not matched to national statistics, not matched to hospital records]

If L_MATCH_TYPE_V=3 & L_MATCH_TYPE_H=3, L_MORB_INCLUDE =0 [child is EUROCAT death only and not found in national statistics, not matched to hospital records]

Children with no hospital admission who died at birth (day 0), L_MORB_INCLUDE =1

Children with no hospital admission who died ≥1 day after birth, L_MORB_INCLUDE =0

## **Appendix 2:**

Guideline for coding confidence in matching variables in different datasets i.e. L_CONFIDENCE, L_CONFID_HDR, L_CONFID_ PX

|  | **All 3 variables available in both datasets for each individual child** | | | | **Only 2 variables present in both datasets**  **for each individual child** | | | | | | | **Only 1 variable available in both datasets for each individual child** | | |
| --- | --- | --- | --- | --- | --- | --- | --- | --- | --- | --- | --- | --- | --- | --- |
| Unique ID | Matched | Not Matched | Matched | Matched | - | Matched | Matched | - | - | Matched | Not matched | Matched | - | - |
| Child’s date of birth | Matched | Matched | Not Matched | Matched | Matched | - | Matched | Matched | Not matched | - | - | - | Matched | - |
| Maternal age | Matched | Matched | Matched | Not Matched | Matched | Matched | - | Not matched | Matched | Not matched | matched | - | - | Matched |
| **Final code** | **Excellent** | **Fair** | **Fair** | **Good** | **Fair** | **Fair** | **Good** | **Poor** | **Not linked** | **Fair** | **Not linked** | **Fair** | **Poor** | **Not linked** |

The “-“ symbol = Not present

If all 3 variables present and matched, code=EXCELLENT

If 2 variables present and both matched, code=GOOD if the 2 variables are unique ID number and child’s DOB

If 2 variables present and both matched, code=FAIR if unique ID number is not one of the 2 variables

If 2 variables present and only 1 matched, code=FAIR if unique ID number is matched

If 2 variables present and only 1 matched, code=POOR if unique ID number is the unmatched variable

If 1 variable present and matched, code=FAIR if it is unique ID number

1. EUROCAT (2013). EUROCAT Guide 1.4: Instruction for the registration of congenital anomalies. EUROCAT Central Registry, University of Ulster [↑](#footnote-ref-1)
2. Please see Figure 1 for data table structure [↑](#footnote-ref-2)
