# Supplementary material for: Creating a population-based cohort of children born with and without congenital anomalies using birth data matched to hospital discharge databases in 11 European regions: Assessment of linkage success and data quality
Source: PLoS One. 2023 Aug 30;18(8):e0290711. doi: 10.1371/journal.pone.0290711 (PMC10468043; doi:10.1371/journal.pone.0290711)
Supplement: S2 Table — (DOCX) [file pone.0290711.s002.docx]

S2 Table. The odds ratio of being included in the study by mother and baby characteristics, by region.

|  | Denmark, Funen | Finland | Italy, Emilia Romagna | Italy, Tuscany | Spain, Valencian Region | UK, Wales | UK, England, East Midlands & South Yorkshire | UK, England, Thames Valley | UK, England, Wessex |  |
| --- | --- | --- | --- | --- | --- | --- | --- | --- | --- | --- |
| Maternal age (years) | | | | | | | | | | |
| 20-34 | 1.00 | 1.00 | 1.00 | 1.00 | 1.00 | 1.00 | 1.00 | 1.00 | 1.00 |  |
| <20 | - | 0.58  (0.48-0.70) | 0.81  (0.74-0.88) | 0.77  (0.43-1.38) | 1.26  (1.18-1.36) | 0.58  (0.48-0.69) | 0.53  (0.46-0.61) | 0.72  (0.51-1.02) | 0.61  (0.49-0.76) |  |
| ≥35 | 0.53  (0.26-1.09) | 1.14  (1.03-1.26) | 0.94  (0.92-0.96) | 1.04  (0.90-1.19) | 0.80  (0.78-0.81) | 0.95  (0.80-1.13) | 1.43  (1.23-1.67) | 1.41  (1.14-1.74) | 1.12  (0.94-1.32) |  |
| Multiple birth status | | | | | | | | | |  |
| Singleton | 1.00 | 1.00 | 1.00 | 1.00 | 1.00 | 1.00 | 1.00 | 1.00 | 1.00 |  |
| Multiple | 0.31  (0.12-0.78) | 0.54  (0.46-0.64) | 0.79  (0.75-0.84) | 1.09  (0.72-1.65) | 1.41  (1.33-1.49) | 0.21  (0.17-0.25) | 1.04  (0.80-1.35) | 0.55  (0.39-0.77) | 0.68  (0.50-0.90) |  |
| Sex | | | | | | | | | |  |
| Male | 1.00 | 1.00 | 1.00 | 1.00 | 1.00 | 1.00 | 1.00 | 1.00 | 1.00 |  |
| Female | 1.42  (0.74-2.74) | 1.10  (1.02-1.18) | 0.92  (0.90-0.94) | 0.84  (0.74-0.97) | 0.85  (0.83-0.87) | 1.32  (1.16-1.49) | 1.00  (0.91-1.11) | 1.03  (0.87-1.22) | 0.95  (0.84-1.07) |  |
| Gestational age (weeks) | | | | | | | | | | |
| ≥37 | 1.00 | 1.00 | 1.00 | 1.00 | 1.00 | 1.00 | 1.00 | 1.00 | 1.00 |  |
| ≤27 | - | 0.03  (0.03-0.04) | 0.32  (0.28-0.38) | 0.33  (0.10-1.07) | 1.68  (1.21-2.33) | 0.005  (0.004-0.006) | 1.68  (0.98-2.87) | - | 0.81  (0.44-1.49) |  |
| 28-31 | - | 0.22  (0.17-0.28) | 0.57  (0.51-0.63) | 0.29  (0.18-0.48 | 2.44  (2.04-2.91) | 0.04  (0.03-0.05) | 0.77  (0.58-1.02) | 0.87  (0.51-1.49) | 0.62  (0.44-0.87) |  |
| 32-36 | - | 0.60  (0.52-0.69) | 1.09  (1.04-1.13) | 0.71  (0.56-0.90) | 1.62  (1.55-1.69) | 0.30  (0.24-0.37) | 0.98  (0.84-1.15) | 0.79  (0.62-1.00) | 0.82  (0.68-0.98) |  |
| Birth weight (grammes) | | | | | | | | | |  |
| 2500-3999 | 1.00 | 1.00 | 1.00 | 1.00 | 1.00 | 1.00 | 1.00 | 1.00 | 1.00 |  |
| <2500 | - | 0.33  (0.30-0.38) | 1.01  (0.97-1.06) | 0.57  (0.46-0.70) | 1.57  (1.51-1.64) | 0.10  (0.09-0.11) | 0.94  (0.82-1.07) | 0.81  (0.66-1.01) | 0.70  (0.57-0.86) |  |
| ≥4000 | 1.28  (0.49-3.33) | 1.53  (1.36-1.73) | 1.21  (1.16-1.26) | 0.95  (0.71-1.25) | 1.14  (1.08-1.19) | 1.15  (0.86-1.53) | 1.13  (0.91-1.41) | 0.81  (0.60-1.09) | 1.07  (0.77-1.50) |  |

-Data not shown due to small numbers
