# Supplementary material for: Creating a population-based cohort of children born with and without congenital anomalies using birth data matched to hospital discharge databases in 11 European regions: Assessment of linkage success and data quality
Source: PLoS One. 2023 Aug 30;18(8):e0290711. doi: 10.1371/journal.pone.0290711 (PMC10468043; doi:10.1371/journal.pone.0290711)
Supplement: S3 Table — (DOCX) [file pone.0290711.s003.docx]

S3 Table. Definition of intensive care in registries with available data

| **Registry** | **Codes used** |
| --- | --- |
| Funen, Denmark* | Procedure codes NABB, NABC, NABE and stays in NICU |
| Emilia Romagna | Local codes for ICU |
| Tuscany | Local codes for NICU, ICU |
| Valencian Region | ICU care identified by local UCI, UCN and UCP codes |
| Finland | Procedure codes WX810, WX870, WX872, WX880, WX890 |
| NNL | Admissions 1995-2011 – local code for specialist providing care 38=neonatology, 52=intensive care internal medicine, 53= intensive care external medicine  Admissions  2013-2017 – local code for specialist providing care 030341=medical specialist intensive care/surgery, 031307= internal medicine intensive care, 031682=paediatric care -neonatology |
| Zagreb | Manual recording of intensive care admission based on hospital records |
| UK, England |  |

NICU = neonatal intensive care unit, ICU = intensive care unit, any type,

* ICU admission defined as child with a procedure code starting with NABA, NABB, NABC or NABE in any admission and/or child registered in neonatal department in first admission [“sengedage_neonatalafdeling_barn” (=bed-days in NICU child)]

A Report of local data sources/content of data available in each registry is available on the EUROlinkCAT website (Milestone 3 Report)
